# 831 Burn Wound Treatment Using Culture Epidermis Allograft

**DOI:** 10.1093/jbcr/iraf019.362

**Published:** 2025-04-01

**Authors:** Hilarion Castañeda, Rocio Muñoz-Sandoval, Elizabeth Lopez

**Affiliations:** Hospital MAC - Star Medica Aguascalientes; Hospital MAC - Universidad Cuauhtemoc; Hospital General de Zona 1, IMSS

## Abstract

**Introduction:**

Cultured epidermis allografts function as biological dressings, promoting the proliferation of the epidermis and facilitating the skin´s self-repair. These have proven effective as a therapeutic option in burn injury patients.

**Methods:**

This is a retrospective, transversal, and descriptive study of acute burn injury patients treated in a private clinic from January 1, 2023, to February 29, 2024. Children and adult patients were included. We reviewed the medical records, including clinical pictures of burn patients treated with culture epidermis allografts.

**Results:**

In total, 34 patients were included; the mean age was 26.5 years, ranging from 1 to 55 years; 65% were male, 35% were women; 26% of the burns were caused by scalding, 68% by direct fire, 3% by electricity, and 3% by mixed burn types; 26% of patients had a superficial second-degree burn, and 74% had a deep second-degree burn.

In 68% of patients, the allograft was applied within 4 hours of the burn; 68% required one application. In all cases, the allograft was applied in the operating room, and all the patients reported decreased pain after the application. None of the patients received antibiotic treatment, and none presented a positive culture test. Likewise, none presented exudate, hyper or hypopigmentation, hypertrophic or keloid scarring, or vascularity. All the patients referred having a satisfactory result.

**Conclusions:**

Culture epidermis allograft is essential in superficial and deep second-degree burns treatment. It decreased pain after the first application, antibiotics were unnecessary, and the patients did not develop an infection; it was always applied in the operating room, under general anesthesia. Complications like exudate, hyper or hypopigmentation, hypertrophic or keloid scarring, or vascularity were not developed. Our young study group stated satisfactory results, promoting a positive body image, function, and most likely adequate social participation in an economically active population group.

**Applicability of Research to Practice:**

Culture epidermis allograft and proper hydration management are effective therapeutic options in the initial care of severe and non-severe burn patients.

**Funding for the Study:**

This program received no specific grant from funding agencies in the public, commercial, or not-for-profit sectors.

